# The Existence of Periodontal Disease and Subsequent Ocular Diseases: A Population-Based Cohort Study

**DOI:** 10.3390/medicina56110621

**Published:** 2020-11-18

**Authors:** Siu-Fung Chau, Chia-Yi Lee, Jing-Yang Huang, Ming-Chih Chou, Hung-Chi Chen, Shun-Fa Yang

**Affiliations:** 1 Institute of Medicine, Chung Shan Medical University, Taichung 40201, Taiwan; cipechau@gmail.com (S.-F.C.); ycb@csmu.edu.tw (M.-C.C.); 2 Department of Ophthalmology, Taichung Tzu Chi Hospital, Taichung 42743, Taiwan; 3 Department of Ophthalmology, Show Chwan Memorial Hospital, Changhua 50093, Taiwan; ao6u.3msn@hotmail.com; 4 Department of Medical Research, Chung Shan Medical University Hospital, Taichung 40201, Taiwan; wchinyang@gmail.com; 5 Department of Ophthalmology, Chang Gung Memorial Hospital, Linkou 33305, Taiwan; 6 Department of Medicine, Chang Gung University College of Medicine, Taoyuan 33302, Taiwan; 7 Center for Tissue Engineering, Chang Gung Memorial Hospital, Linkou 33305, Taiwan

**Keywords:** periodontal disease, eye, keratopathy, inflammation, epidemiology

## Abstract

*Background and objectives:* We aimed to evaluate the correlation between periodontal disease (PD) and following ocular diseases via the National Health Insurance Research Database in Taiwan. *Materials and Methods:* A retrospective cohort study was conducted. Subjects were regarded as having PD according to the diagnostic codes. For comparison, each subject with PD was matched to one non-PD individual from the database after exclusion. The main outcome was defined as the development of infectious keratitis, endophthalmitis, orbital cellulitis, lacrimal duct infection, uveitis and infectious scleritis. Cox proportional hazard regression was used to yield the adjusted hazard ratios (aHR) of ocular diseases between the study and control groups. *Results:* A total of 426,594 subjects were enrolled in both the study and control groups. In the multivariable analysis, significantly higher rates of infectious keratitis (aHR: 1.094, 95% CI: 1.030–1.161), uveitis (aHR: 1.144, 95% CI: 1.074–1.218) and infectious scleritis (aHR: 1.270, 95% CI: 1.114–1.449) were found in the study group. Concerning the PD interval, infectious keratitis (aHR: 1.159, 95% CI: 1.041–1.291) and infectious scleritis (aHR: 1.345, 95% CI: 1.055–1.714) would significantly occur in PD patients with an interval shorter than two years, individuals with a PD interval that ranged from two to five years were under a higher risk of developing uveitis (aHR: 1.184, 95% CI: 1.065–1.315) and infectious scleritis (aHR: 1.386, 95% CI: 1.125–1.708), and the rate of uveitis (aHR: 1.149, 95% CI: 1.038–1.272) was significantly higher if PD persisted more than five years. *Conclusions:* The presence of PD was moderately associated with the risk of developing infectious keratitis, uveitis and infectious scleritis.

## 1. Introduction

Periodontal disease (PD) is a multi-factorial disease that develops in more than 40 percent of the adult United States population [1], which rises to 64 percent in older adults and retards the nutrition status and quality of life significantly [2]. The infection due to the microorganisms in the oral cavity is the main reason for the development of PD [3]. In a more recent study, the inflammatory reaction is regarded as a major mechanism for PD in both the acute and chronic forms [4]. Regarding the management of PD, effective self-care, sodium hypochlorite oral rinse, antimicrobial agents and ultrasonic scalers are applied to eradicate both the infection and inflammation [1].

There are several diseases beyond the oral cavity that may be associated with the existence of PD [5]. Rheumatic arthritis is a disease with autoimmune dysregulation and inflammatory elevation which occurs concurrently with PD according to previous research resulting from the similar inflammation pathway [6,7]. Besides, hypertension is also related to the pre-existing PD with the elevation of both systolic blood pressure and diastolic blood pressure [8]. In addition, infective endocarditis can also be a complication of PD in which pathogens spread via the bloodstream [5,9], and the hyperuricemia status is also correlated to the development of PD [10]. According to the above evidence, PD could influence the whole body due to both the infective and inflammatory reaction.

Previous literature demonstrated the possible relationship between PD and eye disorders [11]. Moreover, a case with endogenous endophthelmitis and severe PD was presented [12]. On the other hand, the management of PD would resolve the ocular morbidity like anterior scleritis [13]. Nevertheless, research with a sufficient study population to survey the correlation between PD and eye diseases is absent. Furthermore, since PD can be chronic and persistent [1], the effect of PD on eye diseases at different times may be altered which has not been fully elucidated.

The purpose of the current study is to evaluate the correlation between PD and subsequent eye disease via the application of the National Health Insurance Research Database (NHIRD) in Taiwan. Furthermore, the distributions of eye diseases in PD with different disease intervals were also analyzed.

## 2. Materials and Methods

### 2.1. Data Source

This population-based, retrospective cohort study was approved by both the Institutional Review Board of Chung Shan Medical University Hospital and the National Health Insurance Administration in Taiwan (Project identification code: CS17075). Moreover, the current study adhered to the declaration of Helsinki in 1964 and its latest amendment. The NHIRD contains the insurance claim data from almost the entire Taiwanese population, as provided by the Taiwan National Health Insurance Administration. Those claim data were collected from the Longitudinal Health Insurance Database 2005 version (LHID 2005) which included records on two million patients who were randomly drafted from the NHIRD at the year 2005 and the whole records of each individual are available from 1 January 2000 to 31 December 2016. For the diagnosis of disease, the International Classification of Diseases, Ninth Revision (ICD-9) and International Classification of Diseases, Tenth Revision (ICD-10) were used for diagnosis of disease in the LHID 2005. In addition to the diagnosis, the basic demography, income level and living region are also available in the LHID 2005 and NHIRD.

### 2.2. Subject Selection

Subjects were defined as PD if the data showed (1) the receipt of diagnosis of PD, and (2) the diagnosis was made by a dentist. For more details, the generally diagnostic criteria of PD including at least one of the following conditions: (1) swollen and bright-red or purplish gums, (2) gums bleeding, (3) a pocket depth deeper than 4 mm, (4) loose teeth, and (5) the loss of periodontal attachment as well as the alveolar bone on X-ray. The index date was regarded as three months after the development of PD; the reason is because a three-month interval is a common treatment period or follow-up interval for periodontal disease in previous research [14,15], thus the periodontal condition as well as the related co-morbidities in other sites may change after a three-month period. To exclude patients with an extremely impaired ocular condition, patients were erased from the study population of the current study if the following conditions occurred: (1) receipt of a diagnosis of legal blindness at any time, (2) receipt of a diagnosis of ocular tumor at any time, (3) receipt of any type of eyeball removal surgery and at anophthalmos status after the index date, (4) the occurrence of the index date before 2005, (5) age younger than 6 years old or older than 100 years old, (6) death of patient before the index date, and (7) any primary outcome (revealed in the following section) developed before the index date. For comparison, each patient with PD was age-, gender- and propensity-score matched (co-morbidities used in propensity-score matching (PSM) are illustrated in the following section) to one individual without PD throughout the study period which constituted the control group. In addition, the study group was divided into different subgroups according to the age, gender and duration of PD interval and compared to corresponding non-PD subjects to evaluate the effect of PD in different conditions.

### 2.3. Main Outcome Measurement

The primary outcomes in the current study were defined as the occurrence of the following infectious or inflammatory ophthalmic diseases after the index date according to the ICD-9/ICD-10 diagnostic codes: (1) infectious keratitis, (2) endophthalmitis, (3) orbital cellulitis, (4) lacrimal duct infection which includes dacryocystitis and caniliculitis, (5) uveitis, and (6) infectious scleritis. To prevent overestimation and confusion, subjects with unclear ocular disease such as “unspecific corneal disorder” or “unspecific disorder of iris and ciliary body” were excluded from the current study. Furthermore, only those who received the diagnosis of the aforementioned ocular diseases by an ophthalmologist were defined as having achieved the primary outcome.

### 2.4. Demographic Variables and Co-Morbidities

To ensure the universal health condition of each subject was as similar as possible and to prevent possible confounders from disturbing the results, the influence of the following parameters were also evaluated in the multivariate analysis model of the current study: age, gender, education level, marriage status, hypertension, diabetes mellitus, ischemic heart diseases, hyperlipidemia, cerebrovascular disease, upper airway infections, oral soft tissue infections, facial cellulitis, rheumatic diseases, liver abscess, allergic otolaryngologic diseases and hordeolum as well as chalazion. We traced the data in the LHID 2005 longitudinally from the index date of each subject to (1) the date when subjects received diagnosis of the last ocular disease, (2) the participant withdrawal from the National Health Insurance schedule, or (3) the end date of NHIRD (31 December 2016).

### 2.5. Statistical Analysis

SAS version 9.4 (SAS Institute Inc., Cary, NC, USA) was used for all the analyses applied in the current study and the statistical methods were similar to a previous study [16]. After age- and gender-matching and PSM with 1:1 proportions of both the study group and control group, the absolutely standardized difference (ASD) was calculated to ensure the distribution of each potential risk factor is homogenous between the study and control groups (an ASD value less than 0.1 is regarded as a similar distribution). Then, a Poisson regression was performed to produce the incidence rate of ocular diseases and the corresponding 95% confidence intervals (CI). In the next step, we used multiple Cox proportional hazard regression to yield the adjusted hazard ratios (aHR) by combining all the demographic information as well as systemic co-morbidities in the analysis model. On the other hand, the subgroup analysis according to the age, gender and disease period of PD in the study group was also conducted. We demonstrated Kaplan–Meier curves to present the cumulative probability of the incidence of ocular diseases between the study and control groups, and then conducted the log rank test to examine whether a significant difference existed between the two survival curves. Since almost all subjects in the NHIRD are from the Han (also known as Chinese) population, ethnicity was not considered as a confounding factor in the current study. Statistical significance was regarded as a *p* value less than 0.05. Because of the calculation method in the statistical software, a *p* value less than 0.0001 was described as *p* < 0.0001.

## 3. Results

### 3.1. Basic Characteristics of Study Population

**Figure 1:**
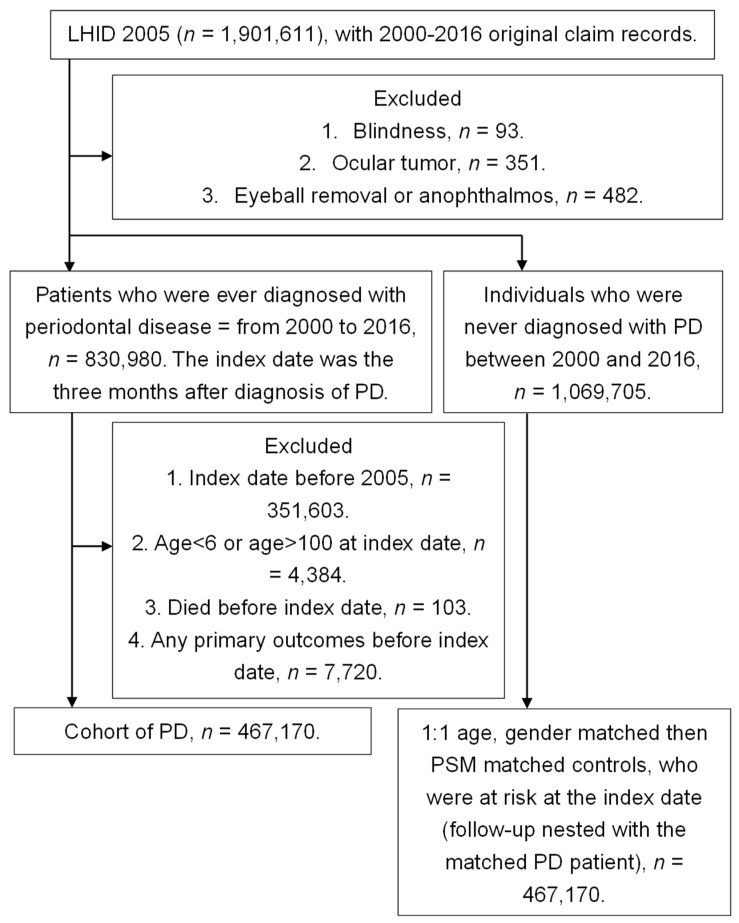
Flowchart of patient selection. PD: periodontal disease, LHID 2005: Longitudinal Health Insurance Database 2005 version, PSM: propensity-score matching.

A total of 426,594 subjects were enrolled in both the study group and the control group, and the flowchart of patient selection is shown in Figure 1. The age, gender, educational level and marriage status showed a similar distribution between the study and control groups according to the ASD. Moreover, the rest of the co-morbidities revealed a similar ratio between the study and control groups, as demonstrated in Table 1.

### 3.2. The Incidence of Ocular Disease in Periodontal Disease

**Figure 2:**
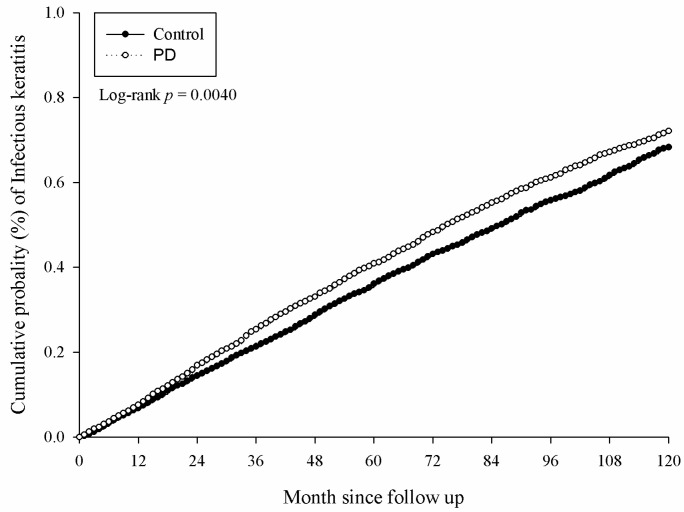
Kaplan–Meier curve for infectious keratitis. PD: periodontal disease.

**Figure 3:**
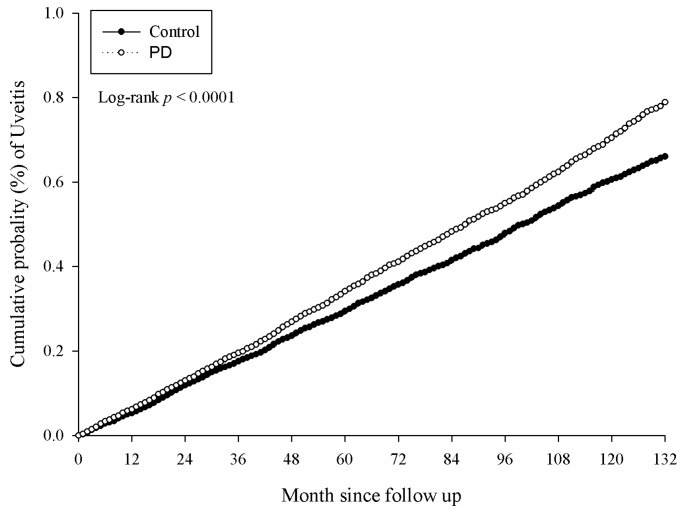
Kaplan–Meier curve for uveitis. PD: periodontal disease.

**Figure 4:**
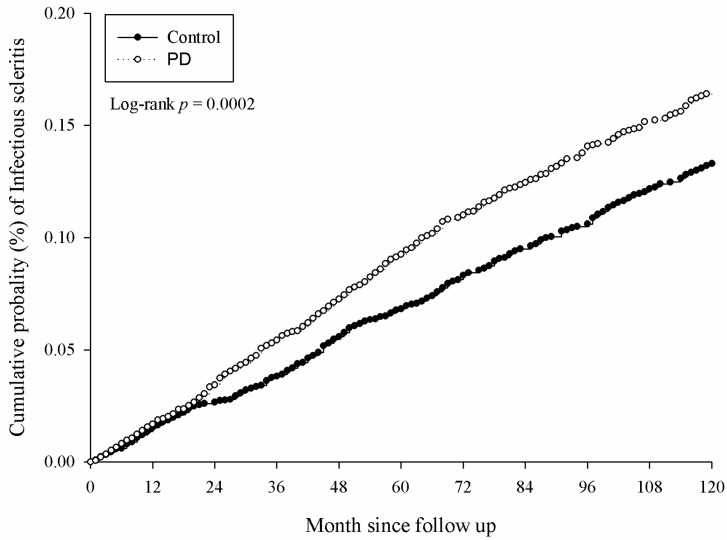
Kaplan–Meier curve for infectious scleritis.

There were 5381 and 4727 events of ocular diseases in the study and the control group, respectively, after a study interval of up to 16 years, and the numbers of each ocular disease are shown in Table 2. After adjusting for multiple potential risk factors including demographic data and co-morbidities mentioned in the above section, significantly higher incidence rates of infectious keratitis (aHR: 1.094, 95% CI: 1.030–1.161), uveitis (aHR: 1.144, 95% CI: 1.074–1.218) and infectious scleritis (aHR: 1.270, 95% CI: 1.114–1.449) were found in the study group compared to the control group (Table 2). Moreover, the significantly higher cumulative probabilities of infectious keratitis (*p =* 0.0040), uveitis (*p =* 0.0001) and infectious scleritis (*p* = 0.0002) were also observed in the study group compared to the control group. The Kaplan–Meier curves of these ocular diseases are demonstrated in Figure 2, Figure 3 and Figure 4.

### 3.3. The Subgroup Analysis for the Development of Ocular Disorders

Regarding the subgroup analysis according to age interval, subjects with PD and aged from 6 to 20 years old showed a higher risk of infectious keratitis development (aHR: 1.148, 95% CI: 1.018–1.294), while subjects with PD and aged from 20 to 39 years showed a significantly higher probability for the development of infectious keratitis (aHR: 1.148, 95% CI: 1.045–1.261), uveitis (aHR: 1.261, 95% CI: 1.114–1.428) and infectious scleritis (aHR: 1.502, 95% CI: 1.212–1.860) in the study group compared to the control group. However, there was no different in the incidence of any ocular diseases between the study and control groups with participants aged 40 and older (Table 3). On the other hand, the male with PD was at higher risk of developing infectious keratitis (aHR: 1.108, 95% CI: 1.006–1.221), uveitis (aHR: 1.204, 95% CI: 1.101–1.317) and infectious scleritis (aHR: 1.335, 95% CI: 1.081–1.649), while females with PD were prone to the influence of infectious keratitis (aHR: 1.081, 95% CI: 1.002–1.166) and infectious scleritis (aHR: 1.228, 95% CI: 1.038–1.453) (Table 4). Regarding the effect of PD disease interval, the infectious keratitis (aHR: 1.159, 95% CI: 1.041–1.291) and infectious scleritis (aHR: 1.345, 95% CI: 1.055–1.714) would significantly occur in those patients with a PD interval shorter than two years, and individuals with a PD interval ranging from two to five years were under a higher risk of developing uveitis (aHR: 1.184, 95% CI: 1.065–1.315) and infectious scleritis (aHR: 1.386, 95% CI: 1.125–1.708). In the population of persistent PD with a disease period longer than five years, only the incidence rate of uveitis (aHR: 1.149, 95% CI: 1.038–1.272) was significantly higher in the study group than the control group (Table 5).

## 4. Discussion

In brief, the current study illustrated the higher incidence of ocular diseases, especially infectious keratitis, uveitis and anterior scleritis, in patients with PD compared to non-PD individuals. Moreover, the PD patients with a younger age were under a higher risk of developing ocular morbidities. Besides, the infectious keratitis and infectious scleritis tend to occur in those with a short-to-middle PD period, while the incidence of uveitis was significantly higher in the population with prolonged PD.

PD is a multi-factorial disease that exhibits a complicated pathophysiology [11,17]. The origin of PD results from the infection of microorganisms and leads to gingival bleeding, loss of attachment and eventually bone destruction [3,18]. There is no major microorganism that contributes to the development of PD while a broad spectrum of commensal microorganisms including bacteria and viruses were found in PD according to previous experiences [19,20]. In severe cases, the delayed healing of the mandibular bone and the distal infection via the bloodstream including the fatal infectious endocarditis, cerebral infarction and myocardial infarction could occur [5,21]. On the other hand, the inflammatory reactions also play an important and complex role in the development and progression of PD in which the proinflammatory cytokine interleukin-17, a major factor of mucosal immunopathology in the oral cavity, accounts for the pathogenesis and progression of PD which are proven in both animal and human studies [4]. Except interleukin-17, other pathways contribute to the pathogenesis of PD including the matrix metalloproteinase 8 which can even serve as a detector for PD [22]. With regard to the ocular lesions, the infectious nature of keratitis, scleritis and certain uveitis has been well-established [23,24,25]. Besides, the elevation of certain inflammatory cytokines like interleukin and matrix metalloproteinase has also been found in ocular diseases including keratitis and uveitis [26,27], and the oxidative stress would also be raised in inflammatory eye diseases [28]. Moreover, the activation of Toll-like receptors was found in both PD and ocular diseases involving the anterior uveitis and keratitis [29,30,31], which implies a similar involvement of innate immunity that could be due to both infection and inflammation. Accordingly, it is possible that the microorganisms involved PD transfer into the ocular region and induce infection via the route of either the cavernous sinus–ophthalmic vein or the anastomosis of anterior ethmoidal artery–posterior ethmoidal artery–sphenopalatine artery, and the elevated inflammation and oxidative stress of PD could influence nearby tissue like the eye. For the potential risk factors, PD and certain ocular diseases share similar systemic co-morbidities such as rheumatoid arthritis [6,24,32]. Besides, the treatment of PD would lead to the resolution of ocular disease in which the scleritis that is refractory to anti-inflammatory management is resolved after the flap procedure and tooth extraction for the co-existing PD [13]. Consequently, PD has the possibility to trigger the development of both the infectious and inflammatory ocular diseases, which is, to some extent, supported by the results of the current study.

PD and the co-existing ophthalmic diseases have been revealed in some studies, mainly the infectious types [12,13,33]. Nevertheless, all of them enrolled less than 10 participants and the strength of the studies is thus diminished. In the current study, the presence of PD appears to lead to a significantly higher incidence of infectious keratitis, uveitis and infectious scleritis development in a study population of nearly one million participants after adjusting for multiple potential risk factors for ocular infection and inflammation. To our knowledge, this is by far the largest study to evaluate the correlation between PD and subsequent ocular diseases considering several confounders. In addition, we excluded those diagnosed with ocular diseases that served as the primary outcome in the current study prior to the index date. As a result, the possible relationship between PD and related ocular morbidities including infectious keratitis, uveitis and infectious scleritis may be illustrated, which is supported via the cumulative probabilities of those diseases. On the contrary, the existence of PD did not significantly increase the chance of endophthalmitis, orbital cellulitis and lacrimal duct infection which is opposite to the case report conducted before [12]. There are two possible explanations for the phenomenon. Firstly, the relatively few outcome numbers of the three diseases, which might lead to some statistical bias because of the low general incidence of them [34,35,36]. Second, the endophthalmitis infectious keratitis would deteriorate to endophthalmitis in some conditions [37]. However, the follow up of one participant was stopped if one of the outcomes was achieved in the current study. As a consequence, although one patient may have had keratitis and then developed endophthalmitis later, only the keratitis episode was recognized and included in the analysis.

In the subgroup analysis, the incidence of infectious keratitis was elevated significantly within two years after PD diagnosis, and the infectious scleritis commonly occurred within five years after PD development. Conversely, the uveitis tended to develop in those with persistent PD that sustained for more than two years. The difference in the occurrence rates among these ocular diseases may result from the natural course of the diseases. Infectious keratitis is an acute condition that often features severe ocular pain, blurry vision and photophobia and patients often seek ophthalmic assessment after just one week [38]. The infectious scleritis, however, shows an indolent course compared to infectious keratitis, and it may be misdiagnosed as conjunctivitis or episcleritis initially in some conditions [25]. Thus, it is possible that the infectious scleritis may be “correctly diagnosed” months or years after initial presentation. Although uveitis can be an acute episode, chronic and recurrent uveitis account for larger proportions than the acute subtype [39]. Moreover, uveitis is related to the local and systemic inflammatory status in a more dominant way than infectious keratitis and infectious scleritis are [39], while the inflammation cytokines are also prolonged and augmented in those with PD [40]. The present evidence may suggest that the inflammation aspect of PD influences the eye more permanently, as supported by the different ocular manifestations with different time periods, which has rarely been illustrated elsewhere.

In terms of the age and gender analysis of PD and related eye diseases, the risk of eye disease development was only prominent in the young population that aged less than 40 years old. The possible reasons for this phenomenon might be that the overall incidence rates of eye disorders were higher in the population younger than 40 in the current study especially for the ocular disease that showed significant difference in the PD group, like keratopathy, and younger patients will search for medical attention more progressively than the elderly according to the clinical experience. On the other hand, the covariates for ocular diseases such as hypertension, diabetes mellitus and ischemic heart disease are commonly found in the elderly, so the effect of these covariates could be considered in the statistical model more frequently in the elderly than in the young and leads to insignificant results. Moreover, the incidence of severe periodontitis with a pocket depth greater than 6mm would reach a peak in patients aged 20 to 40 years old [41], thus a more aggressive PD in juvenile patients may own a greater effect on the development of ocular co-morbidities. For the gender aspect, the trends of the ocular diseases’ development were similar except that the uveitis did not show a significant elevation in the female population compared to the previous findings [39]. However, the lower limit of 95% CI of uveitis in the female population was marginally significant. We speculate that the occurrence of uveitis may also be significant in the female patients of PD after longer follow up periods, although this needs further validation.

On epidemiology, PD is a major disease that affects human health with a prevalence of more than 50 percent in the population older than 65 years and incidence still above 30 percent in those younger individuals [2,42]. The prevalence of PD in the current study was above 20 percent, which was numerically higher than the previous prevalence of PD in Taiwan [43]. In that study, a trend of increasing PD prevalence over recent years was found [43]; the results of the current study correspond with these findings since the time interval of the current study ranged from 2000 to 2016, which is more recent compared to the study period from 1997 to 2013 in the previous study [43]. Since PD influences the majority of the global population and both infectious keratitis and uveitis are the major etiology of legal blindness throughout the world [23,24], ophthalmic examination may be recommended to those with PD to reveal ocular co-morbidity as early as possible to avoid preventable visual impairment.

There are still some limitations in the current study. Firstly, the retrospective design retards the homogeneity of the study and control groups despite the age- and gender-matching and PSM. In addition, the application of claimed data rather than real medical documents in the current study made the evaluation of PD severity and treatment outcome impossible, and may have caused an overestimation of the clinical significance of PD since patients with transient gum swelling may also be regarded as PD in the claimed database. In addition, since putting too many variables in one regression model would lead to the problem of over-fitting [44], we could not enroll all the factors that related to PD and ocular diseases including psoriasis in our multivariable analysis [45,46]. Moreover, the etiology of uveitis cannot be assessed since the ICD-9/ICD-10 system did not separate infectious and noninfectious uveitis. Nevertheless, the characteristics of infectious uveitis also include intraocular inflammation [39], thus the concept that inflammatory processes in PD trigger the uveitis development might still be valid.

## 5. Conclusions

The findings of this retrospective cohort study indicate that PD is associated with moderately increased risks of infectious keratitis, uveitis and infectious scleritis. Patients with newly diagnosed PD seemed to be associated with infectious keratitis while those with chronic PD correlated with uveitis. However, the magnitudes of the associations are low and the findings of the present study may need to be interpreted with caution. Further large-scale prospective studies are warranted to assess the relationship between PD and ocular diseases.

## Figures and Tables

**Table 1:** Characteristics among the study and control groups after propensity score matching.

Basic Characteristics	Study	Control	ASD
*N* (percentage)	426,594	426,594	
Age			0.044
6–20	75,517 (17.70%)	73,288 (17.18%)	
20–39	158,846 (37.24%)	162,049 (37.99%)	
40–59	138,858 (32.55%)	137,982 (32.35%)	
60–100	53,373 (12.51%)	53,275 (12.49%)	
Gender			0.014
Male	196,352 (46.03%)	197,345 (46.26%)	
Female	230,242 (53.97%)	229,249 (53.74%)	
Education			0.050
<6	168,683 (39.54%)	168,293 (39.45%)	
6–9	69,957 (16.40%)	68,450 (16.05%)	
9–12	150,977 (35.39%)	155,830 (36.53%)	
≥12	36,977 (8.67%)	34,021 (7.98%)	
Marriage status			0.000
Not marriage	198,894 (46.62%)	199,868 (46.85%)	
Marriage	188,496 (44.19%)	187,163 (43.87%)	
Divorce	19,525 (4.58%)	19,781 (4.64%)	
Death of spouse	19,679 (4.61%)	19,782 (4.64%)	
Co-morbidities			
Hypertension	43,816 (10.27%)	44,129 (10.34%)	0.002
Diabetes mellitus	20,181 (4.73%)	20,449 (4.79%)	0.003
Ischemic heart diseases	8195 (1.92%)	8208 (1.92%)	0.000
Hyperlipidemia	21,999 (5.16%)	21,607 (5.07%)	0.004
Cerebrovascular disease	6573 (1.54%)	6693 (1.57%)	0.002
Upper airway infections	195,734 (45.88%)	189,686 (44.47%)	0.028
Oral soft tissue infections	4490 (1.05%)	4486 (1.05%)	0.000
Facial cellulitis	256 (0.06%)	279 (0.07%)	0.002
Rheumatic diseases	1574 (0.37%)	1607 (0.38%)	0.001
Liver abscess	53 (0.01%)	51 (0.01%)	0.000
Allergic otolaryngologic diseases	16,831 (3.95%)	15,742 (3.69%)	0.013
Hordeolum and chalazion	3661 (0.86%)	3467 (0.81%)	0.005

ASD: absolutely standardized difference, *N*: number.

**Table 2:** Events of ocular diseases in the study and control groups.

Ocular Disease	Study		Control		aHR (95% CI)
	Person-Months	Event	Person-Months	Event	
Infectious keratitis	35,964,736	2282	35,320,440	2056	1.094 (1.030–1.161) *
Endophthalmitis	36,082,525	143	35,423,178	171	0.799 (0.639–1.007)
Orbital cellulitis	36,080,669	172	35,421,228	193	0.871 (0.709–1.070)
Lacrimal duct infections	36,082,173	151	35,425,872	130	1.101 (0.871–1.393)
Uveitis	35,985,047	2119	35,341,802	1784	1.144 (1.074–1.218) *
Infectious scleritis	36,061,675	514	35,410,360	393	1.270 (1.114–1.449) *

aHR: adjusted hazard ratio adjusted for age, gender, education, marital status, and co-morbidities; CI: confidential interval, * denotes significant difference.

**Table 3:** Events of ocular diseases in different age subgroups.

Ocular Disease	Study		Control		aHR (95% CI)
	Person-Months	Event	Person-Months	Event	
**Age 6–20**					
Infectious keratitis	6,234,633	582	6,071,389	492	1.148 (1.018–1.294) *
Endophthalmitis	6,266,511	4	6,096,636	4	1.003 (0.251–4.013)
Orbital cellulitis	6,264,868	29	6,095,236	31	0.922 (0.556–1.530)
Lacrimal duct infections	6,265,912	10	6,096,682	4	2.424 (0.760–7.731)
Uveitis	6,259,233	143	6,091,721	116	1.204 (0.942–1.538)
Infectious scleritis	6,263,816	53	6,094,741	44	1.159 (0.777–1.729)
**Age 20–39**					
Infectious keratitis	13,593,414	932	13,889,191	827	1.148 (1.045–1.261) *
Endophthalmitis	13,647,328	27	13,934,273	32	0.840 (0.503–1.403)
Orbital cellulitis	13,645,764	58	13,932,632	63	0.928 (0.650–1.327)
Lacrimal duct infections	13,647,781	23	13,935,030	22	1.044 (0.582–1.875)
Uveitis	13,619,287	561	13,912,402	450	1.261 (1.114–1.428) *
Infectious scleritis	13,636,469	210	13,927,604	140	1.502 (1.212–1.860) *
**Age 40–59**					
Infectious keratitis	11,982,844	528	11,575,239	474	1.085 (0.959–1.229)
Endophthalmitis	12,006,899	52	11,597,795	60	0.843 (0.581–1.222)
Orbital cellulitis	12,006,284	56	11,597,317	62	0.875 (0.609–1.256)
Lacrimal duct infections	12,007,033	42	11,598,160	51	0.796 (0.529–1.198)
Uveitis	11,967,214	826	11,562,807	727	1.097 (0.993–1.213)
Infectious scleritis	12,000,242	172	11,592,897	147	1.119 (0.898–1.395)
**Age ≥ 60**					
Infectious keratitis	4,153,845	240	3,784,621	263	0.851 (0.714–1.014)
Endophthalmitis	4,161,787	60	3,794,474	75	0.742 (0.528–1.042)
Orbital cellulitis	4,163,753	29	3,796,043	37	0.736 (0.452–1.197)
Lacrimal duct infections	4,161,447	76	3,796,000	53	1.308 (0.920–1.859)
Uveitis	4,139,313	589	3,774,872	491	1.091 (0.967–1.230)
Infectious scleritis	4,161,148	79	3,795,118	62	1.191 (0.853–1.661)

aHR: adjusted hazard ratio adjusted for age, gender, education, marital status, and co-morbidities; CI: confidential interval, * denotes significant difference.

**Table 4:** Events of ocular diseases in different gender subgroups.

Ocular Disease	Study		Control		aHR (95% CI)
	Person-Months	Event	Person-Months	Event	
**Male**					
Infectious keratitis	16,434,094	872	16,154,942	773	1.108 (1.006–1.221) *
Endophthalmitis	16,477,073	71	16,189,610	89	0.762 (0.558–1.041)
Orbital cellulitis	16,477,026	72	16,189,666	94	0.748 (0.550–1.017)
Lacrimal duct infections	16,478,613	43	16,192,664	36	1.127 (0.723–1.757)
Uveitis	16,425,329	1082	16,150,987	863	1.204 (1.101–1.317) *
Infectious scleritis	16,470,040	206	16,186,651	148	1.335 (1.081–1.649) *
**Female**					
Infectious keratitis	19,530,642	1410	19,165,498	1283	1.081 (1.002–1.166) *
Endophthalmitis	19,605,452	72	19,233,568	82	0.838 (0.610–1.150)
Orbital cellulitis	19,603,643	100	19,231,562	99	0.991 (0.751–1.309)
Lacrimal duct infections	19,603,560	108	19,233,208	94	1.093 (0.829–1.441)
Uveitis	19,559,718	1037	19,190,815	921	1.087 (0.995–1.188)
Infectious scleritis	19,591,635	308	19,223,709	245	1.228 (1.038–1.453) *

aHR: adjusted hazard ratio adjusted for age, gender, education, marital status, and co-morbidities; CI: confidential interval, * denotes significant difference.

**Table 5:** Events of ocular diseases in different periodontal disease interval subgroups.

Event	Study		Control		aHR (95% CI)
	Person-Months	Event	Person-Months	Event	
**PD interval < 2 years**					
Infectious keratitis	9,902,485	719	9852,697	618	1.159 (1.041–1.291) *
Endophthalmitis	9,910,251	43	9859,367	46	0.925 (0.611–1.403)
Orbital cellulitis	9,910,104	44	9859,010	62	0.703 (0.478–1.034)
Lacrimal duct infections	9,910,041	42	9859,397	39	1.055 (0.682–1.632)
Uveitis	9,904,225	557	9854,210	506	1.091 (0.968–1.231)
Infectious scleritis	9,908,949	154	9858,365	113	1.345 (1.055–1.714) *
**PD interval 2–5 years**					
Infectious keratitis	12,683,048	864	12,491,050	776	1.098 (0.996–1.209)
Endophthalmitis	12,718,716	42	12,521,096	63	0.642 (0.434–0.949)
Orbital cellulitis	12,718,150	74	12,520,183	77	0.940 (0.683–1.294)
Lacrimal duct infections	12,718,699	48	12,521,654	34	1.354 (0.872–2.102)
Uveitis	12,691,895	768	12,497,366	629	1.184 (1.065–1.315) *
Infectious scleritis	12,712,409	213	12,517,409	150	1.386 (1.125–1.708) *
**PD interval ≥ 5 years**					
Infectious keratitis	13,379,203	728	12,976,693	705	1.005 (0.906–1.114)
Endophthalmitis	13,453,558	60	13,042,715	65	0.856 (0.603–1.217)
Orbital cellulitis	13,452,415	57	13,042,035	54	1.025 (0.706–1.488)
Lacrimal duct infections	13,453,433	62	13,044,821	57	1.004 (0.700–1.440)
Uveitis	13,388,927	834	12,990,226	681	1.149 (1.038–1.272) *
Infectious scleritis	13,440,317	165	13,034,586	137	1.139 (0.908–1.429)

PD: periodontal disease, aHR: adjusted hazard ratio adjusted for age, gender, education, marital status, and co-morbidities; CI: confidential interval, * denotes significant difference.
